# A Life-Threatening Cause of Stridor, Primary Tracheal Actinomycosis

**DOI:** 10.1590/0037-8682-0195-2025

**Published:** 2026-02-09

**Authors:** Omer Topaloglu, Gokcen Sevilgen, Oguzhan Okcu, Hasan Turut

**Affiliations:** 1 Recep Tayyip Erdoğan University, Faculty of Medicine, Department of Thoracic Surgery, Rize, Turkey.; 2 Recep Tayyip Erdoğan University, Faculty of Medicine, Department of Pathology, Rize, Turkey.

**Keywords:** Tracheal actinomycosis, Biopsy, Rigid bronchoscopy

## Abstract

*Actinomycosis is a chronic, suppurative infection caused by Actinomyces israelii, an anaerobic bacterium that is part of the normal oral flora.* A 47-year-old woman presented to the clinic with dyspnea and sore throat. Computed tomography(CT) revealed thickening of the proximal tracheal lumen and airway narrowing. Pathological examination of biopsy specimens obtained by rigid bronchoscopy revealed periodic acid-Schiff (PAS)(+) bacterial colonization with filamentous bacilli, consistent with *Actinomyces*. This report describes an uncommon presentation of tracheal actinomycosis in which tracheal dilatation was performed for both diagnostic sampling and therapeutic management of tracheal stenosis.

## INTRODUCTION

Actinomyces species are gram-positive, filamentous anaerobes that can cause a wide spectrum of infections, primarily affecting the cervicofacial area, lungs, and abdominal cavity^1^. They are rarely found in the trachea, where infection may become life-threatening^2^. Several predisposing conditions have been associated with the development of actinomycosis, including poor oral hygiene, history of periodontal interventions, chronic alcohol consumption, and smoking-related mucosal injury of the oral cavity or airway^1^. It is two to three times more common in men than in women because of higher incidence of oral trauma and poorer oral hygiene^3^. Considering its rarity, actinomycosis is often overlooked, leading to missed diagnosis. Appropriate anamnesis, history of trauma, clinical and radiological findings, and the presence of *Actinomycetes* sulfur granules in patient samples are crucial for diagnosis. To the best of our knowledge, searches conducted using the Cochrane Library, LILACS, SciELO, MEDLINE, PubMed, and PMC (PubMed Central) revealed no previous reports of tracheal actinomycosis treated with rigid bronchoscopic dilatation and biopsy. This case report presents a rare case of acute tracheal obstruction due to actinomycosis.

## CASE REPORT

**FIGURE 1:**
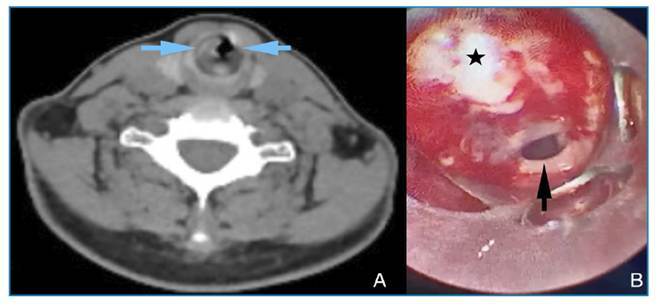
**(A)** Neck CT image showing heterogeneous increased density circularly surrounding the lumen of the proximal trachea and causing mucosal undulation (blue arrows). **(B)** Image showing narrowing of the tracheal lumen (black arrow) by fibrinopurulent tissue (black star) circularly surrounding the trachea during rigid bronchoscopy.

**FIGURE 2:**
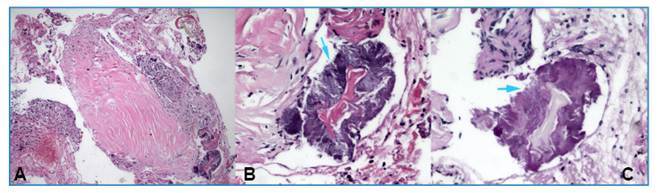
(A) Tracheal tissue showing active-chronic inflammation and bacterial aggregation (H&E × 100). (B) Sulfur granule-like structure composed of centrally located branching gram-positive filamentous *Actinomyces* (indicated by blue arrows) (H&E × 400). (C) Cluster of *Actinomyces* with branching filamentous morphology exhibiting positive reaction to PAS staining (indicated by blue arrows) (PAS × 400).

A 47-year-old woman presented with progressive shortness of breath and cough that had begun 2 months earlier and worsened during the week before admission. She reported throat pain while breathing and had difficulty breathing during admission. The patient had no notable medical history, did not smoke or consume alcohol, and wore a dental prosthesis. On physical examination, stridor was noted, and auscultation revealed prolonged expiration. No pathological findings were detected on the chest radiograph. Chest and neck computed tomography (CT) was planned for further investigation of the cause of stridor. CT imaging revealed a heterogeneous density increase at the subglottic level, reaching a diameter of 5.8 mm in the thickest posterior portion, circularly surrounding the proximal tracheal lumen, and causing corrugation at the mucosal level and narrowing of the airway (Figure 1A). Rigid bronchoscopy was planned for the diagnosis and treatment of tracheal stenosis. Rigid bronchoscopy revealed circular narrowing of the proximal trachea in the subglottic region (Figure 1B). Biopsy samples were obtained from the fibrotic tissue causing narrowing, followed by tracheal dilatation for treatment. The samples were cultured and sent for pathological examination. The patient was discharged the next day with no postoperative complications, and her symptoms had resolved. Pathological examination of the biopsy specimens using hematoxylin and eosin staining revealed reactive squamous epithelial fragments, signs of chronic inflammation, and bacterial infection (Figure 2A and 2B). Periodic acid-Schiff (PAS) histochemical staining revealed filamentous bacilli compatible with *Actinomyces*, which reacted positively (Figure 2C).

**FIGURE 3:**
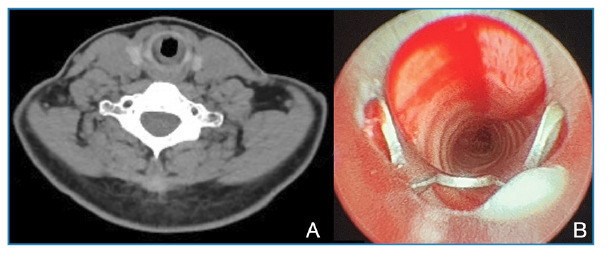
**(A)** Follow-up neck CT scan demonstrating complete resolution of the previously observed tracheal narrowing. **(B)** Follow-up rigid bronchoscopy performed after completion of treatment showing complete resolution of the subglottic tracheal narrowing with restored luminal patency.

Upon receiving the pathology result confirming actinomycosis, the patient was reevaluated by our team to investigate the possibility of an alternative or secondary source of infection. The initial chest CT scan was reviewed, and an additional abdominal CT scan was performed. However, no lesions or findings suggestive of actinomycosis were detected in the lungs, abdomen, or other body regions. In the absence of another demonstrable focus and with all findings confined to the trachea, the case was considered *primary tracheal actinomycosis*. Consequently, a 3-month course of oral amoxicillin (3 g/day) was initiated as targeted antibiotic therapy. Clinical and laboratory responses were detected during the treatment period, and no pathological abnormalities were observed. Follow-up chest and neck CT scans and rigid bronchoscopy performed after completion of treatment demonstrated complete resolution of the tracheal narrowing. The CT revealed normalization of the tracheal lumen (Figure 3A), and bronchoscopy revealed no circular narrowing of the proximal tracheal lumen in the subglottic area, confirming complete healing (Figure 3B). 

## DISCUSSION

Actinomycosis of the larynx or trachea is extremely rare and, therefore, infrequently reported in the literature^4^ ^,^ ^5^. Lesions in the larynx and subglottic regions are often pre-diagnosed as malignant based on their gross appearance^6^. Tracheal actinomycosis can mimic pulmonary infections or malignancies, making diagnosis challenging. Trauma plays an important role in enabling *Actinomyces israelii* to cross the mucosal barrier and enter tissues^3^. Conditions that impair host immunity, including chronic bronchitis, emphysema, diabetes mellitus, cervicofacial trauma, poor oral hygiene, and a history of periodontal procedures, are major predisposing factors for the development of actinomycosis^3^. The patient had no comorbidities but had undergone dental treatment 1 year ago. We believe that dental interventions and periodontal disease may cause trauma and predispose the patient to cervicofacial actinomycosis. 

Dyspnea, cough, and hemoptysis are the most common complaints, frequently accompanied by stridor or recurrent pulmonary infections. A diagnostic delay of 4-6 months after the emergence of symptoms is common. The diagnosis of tracheal actinomycosis may be masked because more common diseases present with similar symptoms^1^. Owing to tracheal lumen stenosis, patients may experience symptoms such as dyspnea, stridor, and difficulty passing secretions. Additionally, the trachea serves as an important reservoir owing to its luminal diameter, suggesting that obstructive symptoms typically appear only after approximately three-quarters of the tracheal lumen are compromised. Owing to this feature, cases are often diagnosed at an advanced stage^7^ ^,^ ^8^. In the present case, the patient had been experiencing progressive shortness of breath over 2 months. It was recently reported that upper airway obstruction may present with inspiratory stridor. This finding suggests that the tracheal lumen gradually narrows over time due to the lesion, and stridor develops once the degree of obstruction exceeds the point of compensation. This is an important symptom of advanced tracheal stenosis. In such cases, failure to implement appropriate precautions and treatments may result in fatal outcomes.

Actinomycosis often mimics other infections and malignancies, and its diagnosis is primarily based on pathological examination because *Actinomyces* does not readily grow in culture^2^. Definitive diagnosis relies on histopathological evidence of granulomatous inflammation with filamentous bacterial colonies and characteristic sulfur granules, together with the demonstration of Actinomyces growth in appropriate tissue cultures. Isolating Actinomyces from sputum or bronchial lavage specimens is often challenging because it requires prolonged anaerobic incubation^1^ ^,^ ^9^. Penicillin remains the first-line therapeutic agent, whereas clindamycin and tetracyclines are effective alternatives for patients with penicillin hypersensitivity^10^. In the present case, a biopsy was performed during rigid bronchoscopy. The specimens showed PAS (+) bacterial colonization, consistent with filamentous bacilli of the Actinomycetes family. The patient was administered oral penicillin for 3 months on an outpatient basis, as hospitalization was declined.

In our case, sustained clinical improvement following antibiotic treatment without recurrence of stridor or airway narrowing strongly supported the diagnosis of tracheal actinomycosis. Unlike benign tracheal stenosis, which typically recurs within 3-4 weeks after mechanical intervention, the patient remained asymptomatic during follow-up. This favorable outcome suggests that the airway obstruction was caused by actinomycotic inflammation and was successfully resolved with appropriate medical therapy.

In conclusion, trachea- actinomycosis is a rare but potentially serious condition that may lead to a delayed diagnosis if not considered in the differential evaluation. Early clinical suspicion, prompt diagnosis, and timely initiation of appropriate treatment can considerably reduce the risk of complications. Diagnosis can be facilitated by clinical awareness and appropriate pathological assessment.

## Data Availability

Research data is only available upon request.
